# Preclinical evaluation of the neutralising efficacy of three antivenoms against the venoms of the recently taxonomically partitioned *E. ocellatus* and *E. romani*

**DOI:** 10.1371/journal.pntd.0013371

**Published:** 2025-08-04

**Authors:** Rebecca J. Edge, Amy E. Marriott, Molly Keen, Tiffany Xie, Edouard P. Crittenden, Charlotte A. Dawson, Mark C. Wilkinson, Wolfgang Wüster, Nicholas R. Casewell, Stuart Ainsworth, Stefanie K. Menzies

**Affiliations:** 1 Centre for Snakebite Research and Interventions, Department of Tropical Disease Biology, Liverpool School of Tropical Medicine, Liverpool, United Kingdom; 2 Department of Infection Biology and Microbiomes, Institute of Infection, Veterinary and Ecological Sciences, University of Liverpool, Liverpool, United Kingdom; 3 Eberhard Karls University of Tübingen, Tübingen, Germany; 4 Molecular Ecology and Evolution at Bangor (MEEB), School of Natural Sciences, Bangor University, Bangor, Wales, United Kingdom; 5 Biomedical and Life Sciences, Lancaster University, Bailrigg, Lancaster, United Kingdom; Universidade Federal do Amazonas, BRAZIL

## Abstract

Snakebite is a significant public health concern in Africa, with the viperid species *Echis ocellatus* being responsible for the majority of snakebite deaths in West Africa. Recently *E. ocellatus* underwent taxonomic revision and was split into two species, *E. ocellatus sensu stricto* and *E. romani*, leading to questions regarding differences in venom bioactivities and the efficacy of antivenoms indicated for treatment of ‘*E. ocellatus*’ envenoming against the two redefined species. Using a range of *in vitro* assays we compared the toxin activities of the two species and the venom-neutralising efficacy of three antivenoms (EchiTAbG, SAIMR Echis and Echiven) raised against ‘*E. ocellatus’*. We then used murine preclinical assays to compare the *in vivo* efficacy of these antivenoms against *E. romani* and *E. ocellatus s. str* venoms. Mitochondrial barcoding of snake skins and venom revealed that *E. romani,* and not *E. ocellatus,* is used in the manufacture of several antivenoms raised against ‘*E. ocellatus*’. There were also a number of differences in specific toxin activity between the venoms of the two species in the three *in vitro* assays utilised in this study.; *E. ocellatus* (Ghana) had the strongest phospholipase A_2_ (PLA_2_) activity, followed by weak PLA_2_ activity for *E. romani* (Cameroon) and insignificant activity by *E. romani* (Nigeria). *E. ocellatus* (Ghana) and *E. romani* (Nigeria) demonstrated comparable snake venom metalloproteinase activity, whilst *E. romani* (Cameroon) had reduced, albeit still significant, activity in comparison. However no differences were observed in a plasma clotting assay measuring coagulopathy between the venoms and localities. Venoms from *E. ocellatus* (Ghana) and *E. romani* (Cameroon and Nigeria) were all recognised comparably by the three antivenoms, and there were only modest differences between antivenoms in neutralising the various *in vitro* toxin effects. In murine preclinical assays, each antivenom could neutralise the lethal effects of *E. romani* (Nigeria), but differences were seen in their comparative potency when the same antivenom doses were tested against *E. romani* (Cameroon) and *E. ocellatus* (Ghana). In these comparative potency assays, all three antivenoms were unable to confer 100% survival when tested against *E. romani* (Cameroon), but SAIMR Echis provided the best protection with 80% survival. When tested against *E. ocellatus* (Ghana), the comparative doses of SAIMR Echis and Echiven provided 100% protection whereas EchiTAbG failed to prevent lethality beyond three hours. This represents the first detailed analysis of differences between *E. ocellatus* and *E. romani* venom bioactivities and the efficacy of existing antivenoms against these two species. Our findings demonstrate that EchiTAbG, SAIMR Echis and Echiven antivenoms are preclinically efficacious against the lethal effects of *E. ocellatus* and *E. romani* venom across a number of localities.

## Author summary

The most medically important snake species in West Africa, *Echis ocellatus,* was recently taxonomically partitioned into *E. ocellatus sensu stricto* and *E. romani*, yet the differences in toxin activities between the venoms and their neutralisation by existing antivenoms remain to be determined. Our phylogenetic analysis revealed several antivenoms were raised against *E. romani* venom rather than *E. ocellatus s. str* venom, and the toxin activities differ between the venoms of these two species. Our data shows that whilst all three tested antivenoms recognised and bound to both *E. ocellatus s. str.* and *E. romani* venoms, there were neutralising differences between the antivenoms in different *in vitro* functional assays, and murine preclinical efficacy assays. These findings provide preclinical evidence and highlight potential differences in the efficacy of three antivenoms in neutralising the preclinical lethal effects of medically important *E. ocellatus s. str.* and *E. romani* venoms. Clinical evidence is required to confirm these findings, but this work suggests that all three antivenoms may be effective against both species despite their recent taxonomic reclassification.

## 1. Introduction

Snakebite envenoming, a World Health Organization (WHO) recognised Neglected Tropical Disease, is estimated to affect 2.7 million people each year, with global annual rates of ~138,000 deaths and over 400,000 people suffering life-altering morbidity [1]. Snakes of the genus *Echis* (common name: saw-scaled or carpet vipers) are one of the most medically important groups of snakes responsible for a large proportion of the global snakebite burden [2], currently consisting of 13 recognised species found throughout much of Africa north of the equator and extending through the Middle East to India and Sri Lanka [3]. Saw-scaled vipers are estimated to be responsible for two-thirds of snakebite envenoming in West Africa [4], with *E. ocellatus* historically invoked as the species responsible for most of the mortality and morbidity from snakebite in this region [2]. Pathophysiological consequences of envenoming by the *Echis* genus are characteristic of viperid snakes, consisting predominately of haemotoxic effects defined by frequent bleeding disturbances, venom-induced consumption coagulopathy (VICC) and local tissue necrosis [5,6].

In 2009, a molecular phylogenetic analysis of the genus *Echis*, including *E. ocellatus* from multiple regions in West Africa, demonstrated the presence of three distinct phylogroups within the species and, given the large degree of morphological variation within the species, led the authors to hypothesise that “additional organismal lineages” may exist [3]. Based on this molecular phylogeny and the analysis of pattern and scalation characters, *E. ocellatus* was partitioned into two species *E. ocellatus* and *E. romani,* in 2018 [7]. The distribution of *E. ocellatus* is now thought to extend from eastern Guinea to north-western Nigeria, while *E. romani* is thought to be resident in northern and north-eastern Nigeria east to at least southern Chad [3,7], with an apparently isolated population in Sudan [8]. For clarity, we will refer to *E. ocellatus* in its old, pre-partition sense, i.e., including *E. romani*, as *E. ocellatus sensu lato*, whereas the post-partition interpretation of *E. ocellatus* will be referred to as *E. ocellatus sensu stricto*.

The only specific therapy for treating snakebite envenoming is antivenom, a polyclonal antibody-based serotherapy generated by immunising large animals (equines/ovines) with crude venom to produce anti-toxin antibodies [9,10]. Currently, there are three antivenoms designed specifically for use against the *Echis* species present in sub-Saharan Africa; EchiTAbG, SAIMR Echis and Echiven, as well as several polyvalent products [11]. The effectiveness of antivenoms in neutralising saw-scaled viper envenoming in Nigeria has been particularly well demonstrated [5,12,13]; EchiTAbG has been robustly examined in controlled trials for clinical efficacy against pre-taxonomic partition *E. ocellatus s.l.* envenoming [5], and SAIMR Echis has good clinical evidence of efficacy [6,14,15]. Echiven is a recent addition to the sub-Saharan African market and to date, to our knowledge, has not been tested in clinical studies or trials and no publicly available preclinical data exist. As of 2020, the efficacy of nine different monovalent and polyvalent antivenoms raised against, or with suggested efficacy via cross-reactivity against, *E. ocellatus s. l.* had been examined in 30 different preclinical studies [16], and the findings of this analysis demonstrated a wide range of reported efficacy, both between the different antivenoms and sometimes for the same antivenom against the same species [12,17].

The recent partition of *E. ocellatus s. l.* means that it is likely that several existing antivenoms indicated for *E. ocellatus* envenoming have been manufactured either using *E. romani* venom or *E. ocellatus s. str.* exclusively, or with a mixture of *E. romani* and *E. ocellatus s.str.* venom. Detailed analyses of the biological differences between *E. ocellatus s.str.* and *E. romani* venom remain outstanding and therefore it remains to be demonstrated whether existing antivenoms have different efficacies in neutralising the venom of each species, although, given their longstanding use against *E. ocellatus s. l.* and known paraspecificity of these antivenoms, it is probable that there will be limited clinical impact of the taxonomic split. Consequently, we sought to determine if the snakes and venoms used to manufacture EchiTAbG antivenom and previous iterations of EchiTab-Plus-ICP antivenom, which were collected in northern Nigeria and maintained at the Liverpool School of Tropical Medicine (LSTM) as part of the EchiTAb study group [12], were indeed *E. ocellatus s. str.,* or *E. romani,* or a mixture of both species. We then investigated the toxin activities of *E. ocellatus s. str.* and *E. romani* venoms using *in vitro* assays, and directly compared the neutralising efficacy of three *Echis* monospecific antivenoms (EchiTAbG, SAIMR Echis and Echiven) in *in vitro* and *in vivo* preclinical assays.

## 2. Methods

### 2.1 Snake specimens and venoms

Skin sheds were obtained from snakes originating from the Kaltungo (Gombe) region of north-eastern Nigeria that were collected between April 2008 and September 2014 and housed in the LSTM herpetarium as part of the EchiTAb study group collection. An additional shed from an *Echis carinatus* (Pakistan) specimen housed at LSTM was also used in this study.

Venom from *E. ocellatus s. l.* (subsequently identified and hereafter referred to as *E. romani* [Nigeria]) was obtained from pooled (N = 48) venom stocks from the EchiTAb study group collection. Pooled venoms of *E. romani* from Cameroon (sold as *E. ocellatus s. l.*, hereafter referred to as *E. romani* [Cameroon]) and *E. ocellatus* from Ghana were purchased from Latoxan, France (Product ID L1114 for both).

### 2.2 Total DNA extraction and Sanger sequencing

Total DNA was isolated from 40 individual snakes in the EchiTAb study group collection. Additionally, a shed skin from one *Echis carinatus* (Pakistan) specimen and lyophilised venoms from *E. ocellatus* (Ghana) and *E. romani* (Cameroon) pooled from individual specimens, purchased from Latoxan (France), were examined. Prior to DNA extraction, sheds were stored individually in zip lock bags at room temperature, then total DNA was isolated using a DNeasy Blood & Tissue Kit (Cat 69504, Qiagen, UK) following the manufacturers recommended tissue extraction protocol for snake sheds (using 2.5 mg of shed) and the blood extraction protocol for resuspended venom (starting with 100 µL of 10 mg/mL venom). Polymerase Chain Reaction of *NADH4* and *CYTB* was performed using primer pairs GludgMod2/EchR for *CYTB* and NADH4/EchR for *NADH4* as described in [7]. Amplicons were Sanger sequenced by Source Bioscience (Cambridge, UK) using respective amplicon primer sets, above. Further details can be found in the S1 Text.

### 2.3 Mitochondrial barcoding and phylogenetic analysis

Resulting sequences were quality checked and aligned using MEGA 11 [18] (RRID:SCR023017). To provide a phylogenetic reference framework, we included in the alignment all sequences of the *E. ocellatus* group (*E. ocellatus*, *E. romani*, *E. jogeri*), sequences of an *E. carinatus* from the United Arab Emirates, and, as an outgroup, sequences of a specimen of *Cerastes cerastes*, which were all sequenced and published as part of a previous phylogenetic study of *Echis* [3]. We implemented the Model function in MEGA 11, using the Bayesian Information Criterion (BIC) to identify the best substitution model for the unpartitioned data prior to Maximum Likelihood (ML) phylogenetic analysis with 100 bootstrap replicates.

### 2.4 Antivenoms and control immunoglobulins

Antivenoms used were; (i) EchiTAbG (whole ovine IgG manufactured by MicroPharm UK Ltd) raised against saw-scaled viper venom of Nigerian origin classified at the time as *E. ocellatus s.l.* (venom provided by LSTM), (ii) snake venom antiserum (Echis) “Echiven” (equine F[ab]’_2_ manufactured by VINS Bioproducts Ltd, India) raised against the venom of saw-scaled vipers from Cameroon, Ghana and Mali (provided by Latoxan, France, all listed as *E. ocellatus*) and (iii) “SAIMR Echis carinatus” antivenom (equine F[ab]’_2_ manufactured by South African Vaccine Producers PTY, South Africa) raised against *E. ocellatus s. l.* from Nigeria and *E. pyramidum* from Ethiopia and Eritrea [19]. An overview of antivenoms used in this study is displayed in Table 1, and evaluation of their protein concentration (by BCA assay) and degradation (by size exclusion chromatography) can be found in S1 Text and S1 Fig. In all subsequent experiments, the volume of antivenom was determined based on the maximum volume capacity permissible for each assay and/or the maximum antivenom volume that did not produce interference in antivenom-only control samples.

**Table 1:** An overview of the antivenoms used in this study. The table shows manufacturer information, batch/lot numbers and expiry dates, composition of antivenom immunoglobulins, protein concentration, and the venom reported to be used in immunisation and their geographical origin (where known) as indicated on the inserts of the products. Asterisk indicates assumed *E. ocellatus sensu lato.* ^ Although stated as *E. carinatus* in the product insert, this now refers to *E. pyramidum* due to taxonomic changes.

Antivenom tested	Manufacturer	Batch/Lot & Expiry date	Characteristics and protein concentration of antivenom	Venoms used for immunisation	Stated efficacy according to product insert
EchiTAbG	MicroPharm Ltd (UK)	- EOG 001440 - January 2017	- Ovine - Liquid - Intact immunoglobulins - 33.8 ± 7.5 mg/mL	E. ocellatus* (Nigeria, snakes maintained at Liverpool School of Tropical Medicine) [5]	*Echis ocellatus*
SAIMR “Echis carinatus”	South African Vaccine Producers (SAVP) PTY, South Africa	- BC 00147 - January 2016	- Equine - Liquid - F(ab’)_2_ fragment of immunoglobulins - 84.8 ± 20.4 mg/mL	E. ocellatus* (Nigeria) and *E. pyramidum* (Eritrea and Ethiopia) [19]	*Echis carinatus^*, *E*. *ocellatus*, *E*. *coloratus*, *Cerastes spp*.
Snake Venom Antiserum (Echis) (“Echiven”)	VINS BioProducts Ltd, India	- 38AS21001 - October 2025	- Equine - Lyophilised powder - F(ab’)_2_ fragment of immunoglobulins - 47.6 ± 5.0 mg/mL	E. ocellatus* (Cameroon, Ghana and Mali, sourced from Latoxan, France) (personal communication)	*Echis ocellatus*

### 2.5 End-point ELISA

Venoms from *E. ocellatus s. str.* (Ghana), *E. romani* (Nigeria), and *E. romani* (Cameroon) were coated at 100 ng per well onto Nunc MaxiSorp ELISA plates (ThermoFisher) for one hour at 37 °C, washed with Tris-buffered saline containing 0.1% Tween20 (TBS-T), and then blocked with 5% milk in TBS-T for two hours at room temperature. Plates were washed in TBST before serial dilutions of each antivenom (diluted in blocking solution) were added to the plate and incubated overnight at 4 °C. The following day, plates were washed in TBS-T and anti-horse or anti-sheep IgG secondary antibodies conjugated to horseradish peroxidase (Sigma) were added at 1 in 1000 dilution in PBS for two hours at room temperature. Plates were washed six times with TBS-T and developed with 0.1 mg/mL ABTS substrate (in 0.05 M citrate buffer pH 5.0 with 0.0075% hydrogen peroxide) for 15 minutes at room temperature. The optical density at 405 nm (OD_405_) was read on a LT-4500 plate reader (Labtech). Control wells consisting of venom-naïve sheep IgG (Biorad) or horse F(ab’)_2_ (prepared from Biorad horse IgG, using Pierce F[ab’]2 preparation kit in accordance with manufacturers instructions) were diluted 1 in 25 or 1 in 5 respectively to match average protein concentration of antivenom 1 in 500 dilutions, then serial diluted as per antivenom. Secondary antibody only control wells were also included.

### 2.6 Phospholipase A2 assay

Neutralisation of venom phospholipase A_2_ (PLA_2_) activity was measured using an EnzCheck Phospholipase A_2_ assay kit (Invitrogen, UK #E10217), as previously described [20]. Optimisation of the amount of venom to be used for each species was first performed to identify the amount of venom that falls within the linear range of enzymatic activity measurements. The relative fluorescence units (RFU) were plotted against the amount of venom per well, and the graphs were manually assessed to identify venom amounts in the linear range of the assay (S2 Fig). The optimal venom amount was determined as 1 µg for all three venoms. Statistical analysis of venom PLA_2_ activity was by ordinary one-way ANOVA performed in Prism 10 (GraphPad, RRID:SCR_00279) and Tukey’s multiple comparison post-hoc test was performed on pairwise comparisons. Antivenoms were serial diluted in PBS containing the pre-defined amount of venom in a 384-well plate and incubated at 37 °C for 30 minutes, following which 12.5 µL PLA_2_ substrate was added to each well. Plates were incubated in the dark at room temperature for 10 minutes and then read at excitation 485–15 nm and emission 520–10 nm. RFU measurements were converted to PLA_2_ activity using the equation of the standard curve, and then expressed as percentage of activity (where the venom only control was 100% activity). For statistical analyses the data were analysed using two-way ANOVA (multiple comparisons) in Prism 9 (GraphPad, RRID:SCR_002798) to compare the antivenoms at each dilution.

### 2.7 Snake venom metalloproteinase assay

Snake venom metalloproteinase (SVMP) activity and neutralisation of the *Echis* venoms was measured using the previously described fluorogenic peptide assay [21], although venom amount was reduced to 500 ng to maintain the same signal window but allow greater resolution between antivenoms and dilutions. Briefly, 500 ng venom was added to wells in a 384-well plate, followed by 10 µL of serial dilutions of antivenom or an equal volume of PBS and incubated at 37 °C for 25 minutes. After cooling to room temperature, 90 µL SVMP substrate solution (ES010 [BioTechne] diluted to 7.86 µM in 150 mM NaCl, 50 mM Tris-Cl pH 7.5) was added to each well (7 µM final well concentration). The plate was immediately read at excitation 320–10 nm and emission 420–10 nm on a plate reader. SVMP activity was calculated for each venom, in which ‘venom only’ wells represent 100% activity and the change in SVMP activity in the presence of the test antivenoms was calculated as a percentage of the ‘venom only’ wells. Ordinary one-way ANOVA was performed in Prism 9 (GraphPad, RRID:SCR_00279) and Tukey’s multiple comparison post-hoc test was performed on pairwise comparisons.

### 2.8 Bovine plasma clotting assay

Plasma clotting activity of the *Echis* venoms and neutralisation by antivenoms was measured using a previously described bovine plasma clotting assay [22,23]. Briefly, 100 ng venom was added to each well in a 384-well plate, followed by 10 µL of serial dilutions of antivenom or an equal volume of PBS. The assay plate was incubated at 37 °C for 25 minutes then room temperature for 5 minutes, before 20 µL of 20 mM calcium chloride (Sigma, #C1016) followed by 20 µL of citrated bovine plasma (Biowest, VWR #S0260) was added to each well. The optical density was immediately read at a wavelength of 595 nm (OD_595_) on a plate reader. For analysis, the cross-section at which the ‘normal plasma clotting’ curve intersected the curves of the test conditions was manually identified and the area under the curve at this time point for each condition was calculated (normalised to venom and PBS only controls) before converting to percentage activity. Ordinary one-way ANOVA was performed in Prism 9 (GraphPad, RRID:SCR_002798), and Tukey’s multiple comparison post-hoc test was performed on pairwise comparisons.

### 2.9. Ethic statement

Animal experiments were conducted under protocols approved by the Animal Welfare and Ethical Review Boards of the Liverpool School of Tropical Medicine and the University of Liverpool, under project licence P24100D38 approved by the UK Home Office in accordance with the UK Animals (Scientific Procedures) Act 1986.

#### 2.9.1 Preclinical experiments.

CD1 mice (male, 6 weeks, 18–20 g, Charles River UK) were grouped in cages of five upon arrival and acclimated for one week before experimentation in specific pathogen-free conditions.

Venom and antivenom were pre-mixed in a volume of 200 µL (diluted in PBS, pH 7.4) and incubated at 37 °C for 30 minutes prior to intravenous injection via the tail vein. Groups of five mice were used as per WHO assay guidelines [9] except in the case of missed or partial doses during injection, as indicated in S6 File. Total numbers used were n = 15 for LD_50_, 61 for ED_50_ and 15 for comparative neutralisation of lethality experiments. Mice were challenged with 5 X LD_50_, using previously reported LD_50_ values of 17.85 µg/mouse for *E. romani* (Nigeria) [24], 33.10 µg/mouse for *E. romani* (Cameroon) [17] and 18.20 µg/mouse *E. ocellatus s. str*. (Ghana) [25].

The median effective dose (ED_50_) assay was performed for *E. romani* (Nigeria) venom to determine the dose of each tested antivenom (µL) that prevented venom-induced lethality in 50% of animals injected with 5 x LD_50_. To compare the neutralising potential of the antivenoms against venoms of *E. romani* (Cameroon) and *E. ocellatus s. str.*, we compared survival rates when animals were administered the dose (volume) of antivenom which prevented lethality in 100% of animals when challenged with 5 x LD_50_ of *E. romani* (Nigeria) venom (control group). Doses used were 100 µL for EchiTAbG, 25 µL for SAIMR Echis and 30 µL for Echiven.

Upon reaching Humane Endpoints (HEPs) or end of experiment, animals were euthanised using rising concentrations of carbon dioxide or cervical dislocation. Time to HEP, number of deaths and number of survivors over the 6-hour experiment duration were recorded as the experimental outcome. ED_50_ values were determined by Probit analysis using StatPlus in Microsoft Excel (AnalystSoft) and results are given as (i) volume of antivenom (µL), (ii) potency (mg/mL) using the equation P = (n – 1 * LD_50_)/ED_50_ where n = number of LD_50_ in the challenge dose [26], and (iii) μL of antivenom required to neutralise 1 mg of venom (µL/mg) [27].

## 3. Results

### 3.1 Establishing the taxonomic identity of captive *E. ocellatus s. str.* by mitochondrial barcoding

**Fig 1:**
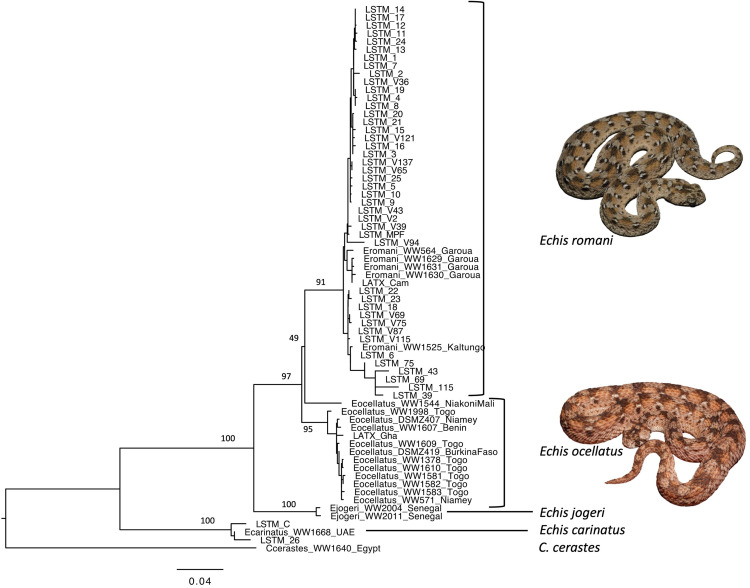
Maximum likelihood phylogeny of the *Echis ocellatus* group inferred from 1433 base pairs of mitochondrial CYTB and NADH4 sequence. Node labels next to major nodes represent %bootstrap support.

To categorically define if the saw-scaled viper venoms used for EchiTAbG and historical EchiTAb-Plus-ICP antivenom production (snakes originating from north-eastern Nigeria, housed in the herpetarium at LSTM) and considered as *E. ocellatus s. l.*, are *E. ocellatus s. str.* or *E. romani*, we implemented a mitochondrial barcoding approach on 40 individuals collected between April 2008 and September 2014. We aligned 789 base pairs (b.p.) of *CYTB* and 644 b.p. of *NADH4* sequence, the aligned fragments corresponding to those used in the previous phylogenetic analysis of the *Echis* genus [3]. The Model function in MEGA 11 identified the Tamura-Nei model [28] with gamma-distributed substitution rates (TN93 + G) as the optimal substitution model under the BIC for the data. Phylogenetic analysis of the *NADH4* and *CYTB* sequences amplified from the shed skins or venoms of the 40 individual Nigerian *E. ocellatus s. l.* specimens in the LSTM collection demonstrate they are all *E. romani* (Fig 1). Similarly, *NADH4* and *CYTB* sequences amplified from *E. ocellatus s. l.* venom originating from Cameroon, sourced from Latoxan, demonstrates clearly it has originated from specimens of *E. romani*, while the sequences amplified from *E. ocellatus s. l.* venom originating from Ghana, sourced from Latoxan, demonstrates this venom has originated from specimens of *E. ocellatus s. str.* (Fig 1). As a control, we also sequenced *NADH4* and *CYTB* amplicons from DNA extracted from the skin shed of the *E. carinatus* specimen originating from Pakistan, with results confirming its *E. carinatus* designation. All the phylogenetic relationships determined were supported by high bootstrap values (>90). These findings strongly suggest that the EchiTAbG and EchiTAb-Plus-ICP antivenoms made using north-eastern Nigerian saw-scaled viper venoms housed at LSTM [5,12], were directed against *E. romani* (EchiTAb-Plus-ICP is now made with ‘*E. ocellatus*’ venom sourced from Latoxan, countries of origin not stated).

### 3.2 Antivenom recognition of *E. ocellatus s. str.* and *E. romani* venoms by ELISA

High levels of comparable binding by all three antivenoms (EchiTAbG, SAIMR Echis and Echiven) was observed against the three venoms *E. ocellatus s. str.*, *E. romani* (Nigeria) and *E. romani* (Cameroon) (S3 Fig). The binding titres remained above naïve control for all antivenoms against all venoms, to at least a 1 in 62,500 dilution of neat antivenom, although this was highest for EchiTAbG. However, there was also a significant difference between the naïve ovine whole IgG and equine F(ab’)_2_ controls, and therefore the apparent slight increase in recognition by EchiTAbG compared to SAIMR Echis or VINS Echis must be interpreted with caution, and may be due differences in the immunoglobulin format and to the differing efficacy of the two secondary antibodies used to detect their target.

### 3.3 PLA_2_ activity and neutralisation by antivenoms

**Fig 2:**
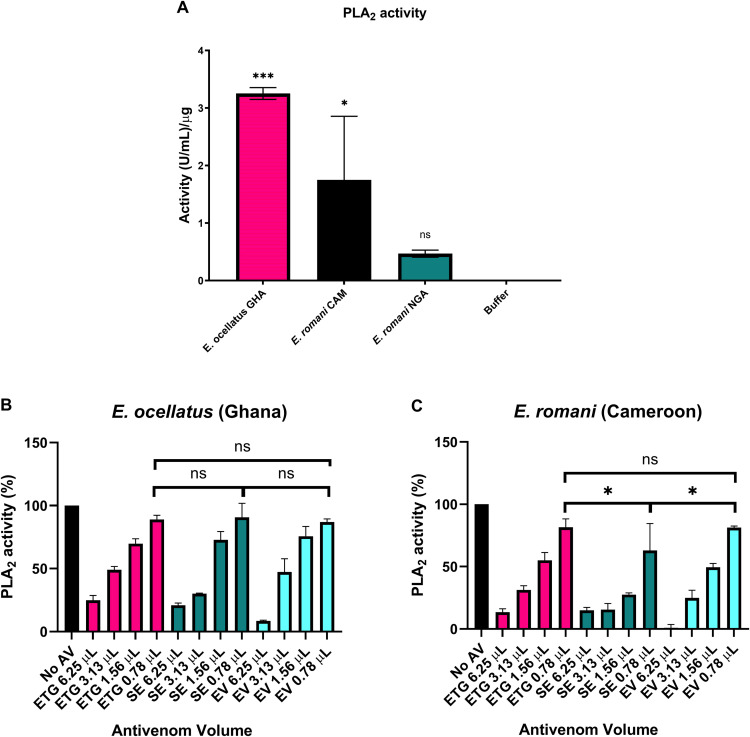
PLA_2_ activity of *E. ocellatus s. str.* and *E. romani* venoms and their neutralisation by the three different antivenoms. A: PLA_2_ activity of E. ocellatus (GHA = Ghana) and E. romani (CAM = Cameroon and NGA = Nigeria). Samples were subtracted for background and converted to activity in (U/mL)/μg by extrapolation from a bee venom standard curve. Data show the mean of three replicates and error bars represent standard deviation. Statistical differences in activity compared to ‘buffer only’ were determined by one-way ANOVA, with venom specific p values indicated above the bar (* indicates p < 0.05, *** indicates p < 0.001, ns = not significant, p > 0.05). B -C: Neutralisation of (B) *E. ocellatus* (Ghana), (C) *E. romani* (Cameroon) PLA_2_ activity by the three antivenoms EchiTAbG (ETG), SAIMR Echis (SE) and Echiven (EV) at different doses, expressed as a percentage of a no-antivenom control showing 100% activity. Data show the mean of three replicates and error bars represent standard deviation. Two-way ANOVA was performed to compare differences in PLA_2_ activity at the 0.78 μL dose of antivenoms. * indicates p < 0.05, ** indicates p < 0.01 *** indicates p < 0.001, ns = not significant (p > 0.05).

The venoms demonstrated moderate differences in enzymatic PLA_2_ activity, as shown in Fig 2A. *E. ocellatus s. str.* activity had the highest activity at 3.25 [U/mL]/µg, compared to 1.75 [U/mL]/µg for *E. romani* (Cameroon), whereas *E. romani* (Nigeria) had negligible activity that was not significantly different to buffer only (p > 0.05). This venom was therefore omitted for further analysis regarding neutralisation. The ability of the three antivenoms to neutralise the *in vitro* PLA_2_ activity of venoms with significant PLA_2_ activity was determined using four different volumes of antivenom (Fig 2B-2C). The three antivenoms demonstrated strong neutralisation of PLA_2_ activity from *E. ocellatus s. str.* venom and Cameroonian *E. romani* venom at the highest amount of antivenom tested (Fig 2B and 2C). At the lowest volume of antivenom (0.78 µL), no significant differences were detected between the antivenoms against *E. ocellatus s. str.* (p > 0.8), whereas for *E. romani* (Cameroon) SAIMR Echis was significantly more effective than EchiTAbG and Echiven (p < 0.015 for both).

### 3.4 SVMP activity and neutralisation by antivenoms

**Fig 3:**
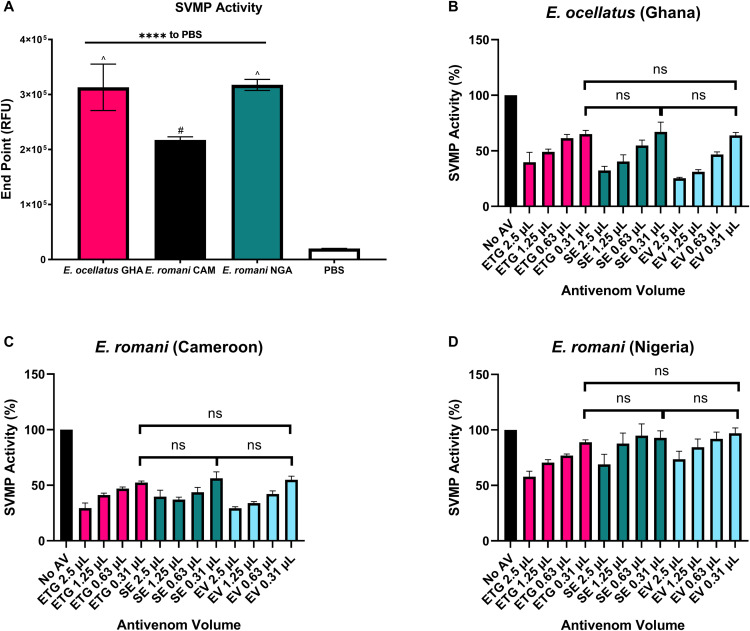
SVMP activity of *E. ocellatus s. str.* and *E. romani* venoms and their neutralisation by the three different antivenoms. A: SVMP activity of *E. ocellatus* (GHA = Ghana) and *E. romani* (CAM = Cameroon and NGA = Nigeria). Data show the mean of four replicates and error bars represent standard deviation. Statistical differences in activity compared to PBS, and between venoms, were determined by one-way ANOVA, with p values against PBS indicated above the bar (**** indicates p < 0.0001), and # indicating significantly lower activity and ^ indicating significantly higher activity. B-D: Neutralisation of (B) *E. ocellatus* (Ghana), (C) *E. romani* (Cameroon) and (D) *E. romani* (Nigeria) SVMP activity by the three antivenoms EchiTAbG (ETG), SAIMR Echis (SE) and Echiven (EV) at different doses, expressed as a percentage of a no antivenom control showing 100% activity. Data show the mean of four replicates and error bars represent standard deviation. One-way ANOVA was performed to compare differences in SVMP activity at the 0.31 μL dose of antivenoms. ns = not significant (p > 0.05).

The *Echis* venoms exhibited strong SVMP activity in the *in vitro* assay compared to PBS control (p < 0.0001), as shown in Fig 3A. *E. ocellatus s. str.* and *E. romani* (Nigeria) venoms had comparable SVMP activity (p > 0.05, denoted by ^ on Fig 3A), and had significantly greater SVMP activity than the *E. romani* (Cameroon) venom (p = 0.0002, denoted by #). The ability of the three antivenoms to neutralise the SVMP activity of the three venoms was determined using four different antivenom volumes (Fig 3B-3D). At 2.5 µL of antivenom, all three antivenoms reduced the SVMP activity of the three venoms by at least 30%, albeit with large variability between venoms and between antivenoms. Indeed, for *E. ocellatus* (Ghana) and *E. romani* (Cameroon), significant reductions were seen by all antivenoms at all volumes tested (p < 0.0001 for all comparisons to no antivenom). However, against *E. romani* (Nigeria), only EchTAbG provided significant reductions beyond the highest antivenom dose (p > 0.05 at 1.25 µL for both SAIMR Echis and Echiven). At the lowest volume tested (0.313 µL), there were no significant differences between antivenoms against each of the venoms, but they were all less effective against *E. romani* (Nigeria) with reductions of less than 12% observed (Fig 3D).

### 3.5 Plasma clotting activity and neutralisation by antivenoms

**Fig 4:**
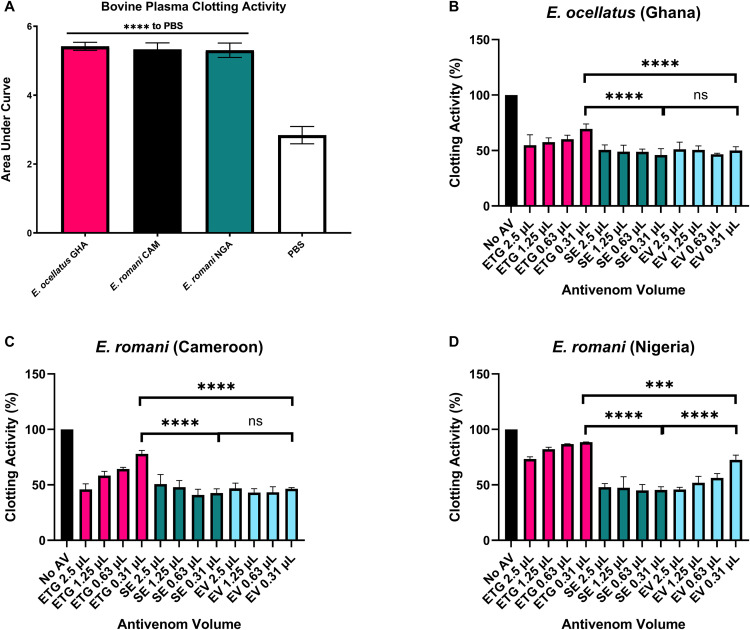
Plasma clotting activity of *E. ocellatus s. str.* and *E. romani* venoms and their neutralisation by the three different antivenoms. A: Plasma clotting activity of *E. ocellatus* (GHA = Ghana) and *E. romani* (CAM = Cameroon and NGA = Nigeria). Data show the mean of four replicates and error bars represent standard deviation. Statistical differences in activity compared to PBS, and between venoms, were determined by one-way ANOVA, with p values against PBS indicated above the bar (**** indicates p < 0.0001). B-D: Neutralisation of (B) *E. ocellatus* (Ghana), (C) *E. romani* (Cameroon) and (D) *E. romani* (Nigeria) plasma clotting activity by the three antivenoms EchiTAbG (ETG), SAIMR Echis (SE) and Echiven (EV) at different doses, expressed as a percentage of a no antivenom control showing 100% activity. Data show the mean of four replicates and error bars represent standard deviation. One-way ANOVA was performed to compare differences in plasma clotting activity at the 0.31 μL dose of antivenoms. **** indicates p < 0.0001, *** indicates p < 0.001, ns = not significant (p > 0.05).

Each venom possessed a significant ability to cause clotting of bovine plasma compared to PBS control (p < 0.0001), with comparable activity between the venoms (p > 0.05, Fig 4A). When tested at 2.5 µL per well, each antivenom was able to reduce clotting activity against all three venoms, however, the percentage of clotting activity remaining was variable, particularly for antivenoms against the same venom. Whilst SAIMR Echis and Echiven were able to significantly reduce coagulopathic activity to approximately 50% for all three venoms (p < 0.0001 for all antivenom volume comparisons to no antivenom), EchiTAbG could only achieve this against *E. ocellatus s. str.* and *E. romani* (Cameroon) with declining significant reductions against *E. romani* (Nigeria) (p = 0.0034 at 0.63 µL and p = 0.017 at 0.313 µL). At the lowest volume of antivenom tested (0.313 µL), there were significant differences between the effectiveness of the antivenoms to modulate clotting activity. SAIMR Echis was significantly better at reducing the clotting activity of the Nigerian locality of *E. romani* than Echiven or EchiTAbG (reduced to < 50% vs 70–90% respectively, p < 0.0001) (Fig 4D), whilst against the *E. ocellatus* (Fig 4B) and Cameroon locality (Fig 4C), SAIMR Echis and Echiven were both significantly better than EchiTAbG (~50% compared to ~70%, p < 0.0001).

### 3.6 Ability of antivenoms to neutralise murine venom induced lethality

The antivenom efficacies and potencies were determined for each antivenom against the venom of *E. romani* (Nigeria) and are presented in Table 2. These experiments demonstrated notable differences in the capacity of antivenoms to neutralise *E. romani* (Nigeria) venom induced lethality. By volume of antivenom and potency, SAIMR Echis was the most efficacious antivenom. In comparison, Echiven was two-fold less potent and EchiTAbG was seven-fold less potent.

**Table 2:** Antivenom efficacy against *E. romani* (Nigeria) in a murine pre-incubation model. The efficacy of EchiTAbG, SAIMR Echis and Echiven against *E. romani* (Nigeria) reported as ED_50_ (µL), potency (mg/mL) and volume of antivenom per mg of venom (µL/mg)*.* 95% confidence intervals indicated in parentheses.

*E. romani *(Nigeria)	EchiTAbG	SAIMR Echis	Echiven
**ED**_**50**_ **(µL)**	50.0 (5.7 –130.0)	7.1 (0.5 – 44.4)	12.7 (5.0 – 31.2)
**Potency** **(mg venom/mL antivenom)**	1.4 (0.6 – 12.6)	10.1 (1.6 – 152.5)	5.6 (2.3 –14.4)
**Volume of antivenom per mg venom (µL/mg)**	560.5 (63.7 – 1456.5)	79.1 (5.3 – 497.0)	142.0 (55.8 – 349.6)

We next implemented a comparative neutralisation of lethality assay, similar to one previously utilised for comparing the efficacy of various polyvalent antivenoms for East Africa [29]. We examined the ability of each antivenom to neutralise 5 x LD_50_s of *E. romani* (Cameroon) and *E. ocellatus s. str.* venom compared to a fixed venom dose - the volumes of antivenoms that conferred 100% survival against Nigerian *E. romani* venom (EchiTAbG 100 µL, SAIMR Echis 25 µL and Echiven 30 µL).

**Fig 5:**
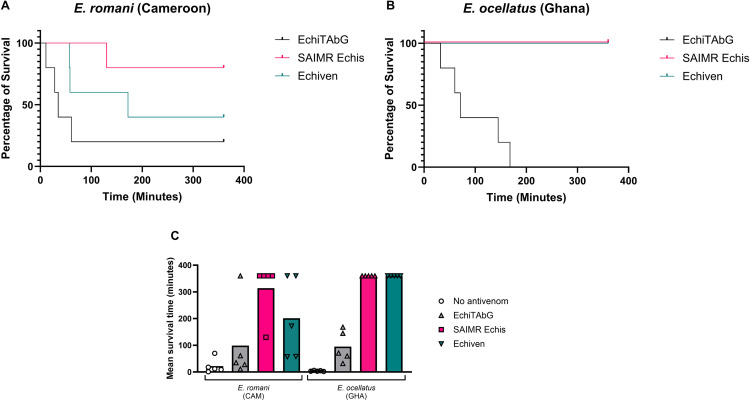
ED_100_ survival times. A: *E. ocellatus* (Ghana) B: *E. romani* (Cameroon) C: Mean survival time of animals – bars indicate mean survival time and markers indicate individual survival times for each animal. Each experiment used five mice per dose group, challenged with a dose of 5 x venom LD_50_ and monitored for 6 hours. EchiTAbG shown in grey, SAIMR Echis shown in magenta, Echiven shown in teal.

All three antivenoms provided only partial protection against 5 x LD_50_ of *E. romani* (Cameroon) venom at these doses (SAIMR Echis 80% survival, Echiven 40% survival, and EchiTAbG 20% survival) (Fig 5A). When using the same doses against 5 x LD_50_ of *E. ocellatus* (Ghana) venom, both SAIMR Echis and Echiven provided complete protection, with 100% of mice surviving until the end of experiment (Fig 5B). In contrast, EchiTAbG failed to prevent lethality but further increased time to humane endpoints (Fig 5C, mean survival times 95 minutes, compared to 6 minutes with no antivenom).

## Discussion

Given the medical significance of envenoming by snakes of the genus *Echis* [30], the identification of efficacious antivenoms suitable to treat such snakebites is integral to the WHO’s objectives to halve snakebite mortality and morbidity by 2030 [31]. This preclinical study aimed to test available *Echis*-specific monospecific antivenoms and directly compare their ability to neutralise *E. ocellatus sensu lato* venoms, both *in vitro* and in preclinical murine models of envenoming. This is particularly important considering the recent changes in taxonomy to the genus which has seen *E. ocellatus*, historically viewed as the most medically important species of the genus in Africa [6], split into *E. romani* and *E. ocellatus s. str.* [7]. The recent taxonomic change raised important questions regarding differences in venom composition between the newly identified *E. romani* and *E. ocellatus*, and naturally raised questions around potential efficacy of antivenoms indicated for *E. ocellatus* pre-species partition, which we sought to address in this study.

The saw-scaled vipers housed in the LSTM herpetarium that were barcoded in this study all originate from the Kaltungo (Gombe) region of north-eastern Nigeria. The barcoding results presented here clearly demonstrate that all these animals, historically considered *E. ocellatus s. l.* prior to partition, are *E. romani*, further evidencing the apparent distinct geographical ranges of the newly partitioned species [3,7]. Furthermore, based on the genetic barcoding results presented, it is likely that several existing antivenoms indicated for *E. ocellatus* envenoming are highly likely to have been manufactured using a mixture of *E. romani* and *E. ocellatus* venom or solely *E. romani* venom. The latter will certainly be the case with EchiTAbG and previous iterations of the trivalent antivenom EchiTAb-Plus-ICP, which have been manufactured using the venom of some of the snakes barcoded in this study [12].

The main proteinaceous components of *Echis* venoms are SVMPs, PLA_2_, C-type lectin-like proteins, serine proteases, disintegrins, and L-amino acid oxidases [17,32,33]. In particular, the *Echis* genus of snakes have a remarkably high abundance of SVMPs, and specifically for *E. ocellatus/romani* venoms which comprise up to 70% of the venom [34]. These zinc-dependent proteinases play a fundamental role in driving venom-induced consumption coagulopathy and systemic haemorrhage [35]. The *in vitro* SVMP activity of each of the three venoms were significantly neutralised by the highest dose of each antivenom. With regards to PLA_2_, a previous transcriptome analysis of various *Echis* species [36] demonstrated proportional differences in relative Group II PLA_2_ abundance across different species, with markedly less detected in *E. romani* (previously *E. ocellatus*) [36]. Similarly, venomic analyses of *E. romani* (previously *E. ocellatus*) from different locales demonstrated intraspecies differences in the abundance of PLA_2_ [37], and this was demonstrated by evident differences observed in our *in vitro* PLA_2_ assay. Bearing in mind the known variation in venom toxins and subsequent activity, this reiterates the importance of carefully evaluating the source of venoms used for antivenom production and the need for thorough and transparent preclinical testing of proposed species efficacy. Our ELISA results demonstrated little to no difference with regards to the ability of each antivenom to recognise each venom, and suggests that, for *Echis* species at least, this may not be an appropriate method for estimating neutralising capabilities given the differences observed in the functional *in vitro* assays.

Whilst *in vitro* assays are an important tool to identify potential efficacious snakebite treatments, preclinical efficacy testing remains heavily reliant on murine neutralisation of lethality assays due to the complexity and multiplicity of venom activities *in vivo*. The *in vivo* efficacy results presented here demonstrate that each *Echis* monospecific antivenom was capable of neutralising the lethal effects of Nigerian *E. romani*, however notable differences are seen in their comparative preclinical potency against venoms from *E. ocellatus* and other geographic locales of *E. romani*. The calculated ED_50_ values were in broad agreement with previously calculated ED_50_ values for EchiTAbG and SAIMR Echis against Nigerian *E. romani* (formerly *E. ocellatus*) venom [38], with SAIMR Echis possessing the most potent venom-neutralising ability. When accounting for total protein concentration of the three *Echis* specific antivenoms (Table 1), the differences in neutralising efficacy between products were more modest, although the trends in neutralising ability remained. In addition to total protein content, the antivenoms also had different formulations which can be difficult to control for. However, previous studies comparing whole IgG and F(ab)’_2_ against envenomation by *Bothrops asper* found no significant differences between the two formats [27,39].

When the antivenoms were assessed further for cross-reactivity, intra-country and intra-species differences became apparent. The dose of each antivenom that neutralised 100% of lethality against *E. romani* (Nigeria) was unable to fully protect mice challenged with *E. romani* venom from Cameroon, whilst SAIMR Echis and Echiven fully prevented lethality from *E. ocellatus s. str.,* but EchiTAbG could not at the dose tested. The results mirrored the *in vitro* and ED_50_ findings, with substantial differences in dose-matched potency of antivenoms against other species. The most notable finding was the lower ability of antivenoms to protect mice from *E. romani* (Cameroon) envenoming when using a protective *E. romani* (Nigeria) antivenom dose, with 60% and 80% of mice succumbing to venom effects when dosed with Echiven and EchiTAbG, respectively. This suggests that *E. romani* venoms from different localities have different potencies and thus differences in their ability to be neutralised by antivenoms, meriting further research to understand the impact of intraspecific *E. romani* venom variation on antivenom efficacy [40].

These findings illustrates how, given the frequency of sometimes extreme venom variation within species, even in the face of extensive gene flow [41], taxonomic revisions should be seen as broad roadmaps for additional research into antivenom efficacy, but not interpreted as robust predictors of venom composition or antivenom effectiveness [42]. In view of the public health importance of the *E. ocellatus* complex, further research into variation in venom composition within the group would be advisable. It is also important to note here that only a single dose has been examined to enable comparative analysis of the ability of an antivenom to neutralise lethal pathology of different venoms at that dose, and results should therefore be viewed in this context and treated with caution and not extrapolated to the human scenario. EchiTAbG has been proven to be clinically effective in

Nigeria [5,11,12], and has a WHO positive risk-benefit assessment [43] for treatment against *E. ocellatus* from Ghana, *E. romani* from Cameroon and *E. pyramidum* venoms from Egypt and Kenya (personal correspondence). Whilst previous polyvalent products produced by VINS for use in Africa have not proven efficacious in independent testing [29,37] our murine model of systemic envenoming demonstrated both SAIMR Echis and the sample of Echiven provided by VINS had relative superior dose efficacy for all venoms investigated compared to EchiTAbG.

In this study we chose to implement a comparator model of envenoming – where animals are given the same dose of antivenom against different venoms and survival is compared - to reduce the number of animals required to compare whether the antivenoms demonstrated similar preclinical neutralising efficacy against closely related venoms. Such comparator models of envenoming therapy efficacy have been used successfully in the past to discriminate between different antivenom efficacies whilst substantially reducing the number of animals and capital cost required as compared to standard ED_50_ testing [29]. Due to their inherent limitations, such models cannot provide direct comparison of the neutralising potency of different antivenoms against a particular venom, as can ED_50_ experiments, but they do allow inference of potential efficacy and limitations in neutralising potential at a single comparable dose. The comparison of expired and non-expired antivenom could be considered as a limitation. However, the efficacy of antivenoms has been demonstrated to be maintained decades after expiry [44–46], and therefore we are confident the use of expired antivenoms has had minimal to no impact on this study. Additionally, we used SEC analysis of the antivenoms (see S1 File) which demonstrated most of the protein content in each antivenom sample was of the expected size, i.e., non-degraded.

In summary, all antivenoms conveyed a degree of intra-genus preclinical neutralisation amongst the sub-Saharan African *Echis* venoms, although this was highly variable across the different *Echis* species and the three *Echis* monospecific antivenoms tested. Ultimately, our data provides the first empirical evidence of differences in venom potencies and antivenom efficacies against the recently partitioned medically important west African saw-scaled viper species *E. romani* and *E. ocellatus.*

## Supporting information

S1 Text Contains further methodological detail on: venom storage, DNA extraction (from venoms and skins), PCR, Sanger sequencing, antivenom reconstitution and control immunoglobulins, size exclusion chromatography, ELISA, PLA2 assay, SVMP assay, plasma clotting assay, preclinical assays. (DOCX)

S1 File BCA assay raw data. (XLSX)

S2 File ELISA raw data. (XLSX)

S3 File PLA2 assay raw data. (XLSX)

S4 File SVMP assay raw data. (XLSX)

S5 File Plasma clotting assay raw data. (XLSX)

S6 File Preclinical assays survival times. (XLSX)

S1 Fig Size exclusion chromatography (SEC) analysis of the three antivenoms and control proteins. Analysis was carried out using a Superdex 200 SEC column equilibrated in PBS and elution was monitored at 214 nm. Fifty μL (50 μg) of sample was loaded. The native molecular weights (kDa) of the proteins are indicated above the respective peak.: SEC analysis of (A) control immunoglobulins whole sheep IgG (blue) and equine F(ab’)_2_ (gold), (B) whole sheep IgG (blue) and EchiTAbG (gold) and (C) equine F(ab’)_2_ (blue), SAIMR Echis (gold) and Echiven (‘VINS’, green).
(TIF)

S2 Fig PLA_2_ assay optimisation. Graphs show the fluorescence intensity measured in the EnzCheck PLA_2_ assay with different amounts of each venom. Amounts of venom that fall in the linear range were used for subsequent assays of venom PLA_2_ neutralisation by antivenoms. *E. ocellatus* (Ghana) shown in magenta, *E. romani* (Cameroon) shown in black and *E. romani* (Nigeria) shown in teal. Samples were subtracted for background fluorescence. Data show the mean of three replicates and error bars represent standard deviation.
(TIF)

S3 Fig The titre of three antivenoms against *E. ocellatus s. str.* and *E. romani* venoms determined by end-point titration ELISA. EchiTAbG shown in grey, SAIMR Echis shown in magenta, and Echiven shown in teal. Venom-naïve equine F(ab)’_2_ shown in dark purple and venom-naïve ovine IgG shown in light purple. Panel A: *E. ocellatus* (Ghana). Panel B: *E. romani* (Cameroon). Panel C: *E. romani* (Nigeria). Data points represent the mean of two replicates and error bars show the standard deviation.
(TIF)
