# Supplementary material for: Preclinical evaluation of the neutralising efficacy of three antivenoms against the venoms of the recently taxonomically partitioned E. ocellatus and E. romani
Source: PLoS Negl Trop Dis. 2025 Aug 4;19(8):e0013371. doi: 10.1371/journal.pntd.0013371 (PMC12321083; doi:10.1371/journal.pntd.0013371)
Supplement: S1 Text — (DOCX) [file pntd.0013371.s001.docx]

**Supplementary Methods**

Venoms

Venoms were stored as lyophilised powders at 4 °C until reconstitution, and were reconstituted in PBS pH 7.4 (Gibco, UK #10010) to 10 mg/mL stocks and aliquoted for storage at -80 °C.

Total DNA extraction, PCR and Sanger sequencing

The DNA was sourced from individual skin sheds for those snakes currently held in captivity at the time of this study, or historical lyophilised venom samples extracted from individual snakes previously held in the collection. Venoms were resuspended in PBS pH 7.4 (Gibco, UK #10010) prior to DNA extraction. Total DNA was isolated using a DNeasy Blood & Tissue Kit (Qiagen, UK, #69506) following the manufacturers recommended “tissue extraction protocol” for shed snake skin (using ~2.5 mg of skin) and the “blood extraction protocol” for resuspended venom (starting with 100 µL of 10 mg/mL w/v venom). Resulting amplified DNA was subsequently purified using a QIAquick PCR Purification Kit (Qiagen, UK #28104) and quality checked by 1% agarose (Severn Biotech, UK #30-10-10) gel electrophoresis (180 V, 30 minutes) in 1 X Tris-acetate EDTA buffer (Severn Biotech, UK #20-6001-10, final composition 40 mM Tris-acetate, 10 mM EDTA, pH 8.0).

at a final concentration of 0.5 µM per primer with Phusion Green Hot Start II High-Fidelity PCR Master Mix (ThermoFisher UK, #F566S). Approximately 1 ng of template DNA was used per reaction. PCR was performed on a 5PRIMEG/02 or TC-512 thermocycler (both Techne [Cole-Parmer], UK), with the following cycle conditions: initial denaturation (98 °C, 30 s), followed by 30 cycles of denaturation (98 °C, 10 s), annealing (50 °C, 10 s) and extension (72 °C, 15 seconds), followed by a final extension step (72 °C, 300 s). Total DNA extracted from venom solutions was typically very low yield (<1 ng/µL), resulting in poor PCR amplification of both markers. To obtain suitable yields of DNA for sequencing, reactions were purified using a QIAquick PCR Purification Kit (Qiagen, UK #28104) and eluted material used as a template in a second round of PCR using the same conditions as the first round.

Antivenoms

Antivenoms EchiTAbG and SAIMR Echis were donated to Liverpool School of Tropical Medicine by UK health authorities post expiry, whilst a sample of Echiven was kindly donated by VINS Bioproducts Ltd. The lyophilised Echiven was resuspended in 10 mL sterile water (provided by the manufacturer) prior to use. Control equine F(ab)’_2_ for incorporation as a control in the *in vitro* experiments described below was produced from equine IgG (BioRad #PEP001) using the Pierce F(ab)’_2_ Preparation Kit (Pierce, ThermoScientific #44988) according to manufacturer’s protocols.

Antivenom protein concentration was determined using a Pierce BCA Protein Assay kit (ThermoFisher, #23225), using known concentrations of purified ovine and equine IgG (BioRad #PSP01 and #PEP001, respectively) to produce ovine and equine IgG standard curves. To determine the protein concentration of EchiTAbG, data were interpolated from the ovine IgG standard curve, and to determine the protein concentration of SAIMR Echis and Echiven, data were interpolated from the equine IgG standard curve. The BCA assay was performed according to the manufacturer’s protocols using the microplate assay with an incubation at 37 °C for 30 minutes, with each test condition tested in duplicate. All three antivenoms were diluted 1 in 100 and 1 in 200 in PBS (Gibco, #20012, pH 7.4) before assaying, to dilute the antivenoms to fall within the standard curve and working range of the BCA assay, and the mean protein concentration estimate from the two dilutions was determined in Excel (Microsoft, RRID:SCR_016137).

Size exclusion chromatography

Analysis was carried out using a 24 mL Superdex 200 SEC column operating at 0.5 mL/min in 25 mM sodium phosphate, 0.15 M NaCl, pH 7.2. Elution was monitored at 214 nm. Fifty μL (50 μg) of sample was loaded. The column was calibrated using SEC standards run under the same conditions (BioRad: ferritin, IgG, ovalbumin, myoglobin, cyanocobalamin). Immunoglobulins run under the same conditions for comparison were whole ovine IgG (Biorad #PSP01) and equine F(ab’)_2_ digested from BioRad #PEP001 using Pierce F(ab’)_2_ Preparation Kit (ThermoFisher, #44988) in accordance with the manufacturers instructions.

ELISA

Venoms from *E. ocellatus s. str.* (Ghana), *E. romani* (Nigeria), and *E. romani* (Cameroon) were coated at a concentration of 100 ng per well onto Nunc MaxiSorp ELISA plates (ThermoFisher) in 50 mM carbonate-bicarbonate coating buffer pH 9.5 (Sigma #C3041) and allowed to bind for one hour at 37 °C. Plates were washed six times with Tris-buffered saline with 0.1% Tween20 (TBS-T), and then blocked with 5% milk in TBS-T for two hours at room temperature. Plates were washed three times in TBST before each antivenom (neat, non-normalised) was diluted 1 in 500 in blocking solution, added to the plate and five-fold serial diluted six times before being incubated overnight at 4 °C. The following day, plates were washed six times in TBS-T and anti-horse or anti-sheep IgG secondary antibodies conjugated to horseradish peroxidase (Sigma #A6917 and #A3415, respectively) were added at 1 in 1000 dilution in PBS for two hours at room temperature. Plates were washed six times with TBS-T and developed with ABTS substrate (0.1 mg/mL 2,2’-azino-bis[3-ethylbenzthiazoline-6-sulfonic acid] diammonium salt [Sigma #A9941] in 0.05 M citrate buffer pH 5.0 with 0.0075% hydrogen peroxide) for 15 minutes at room temperature. The optical density was immediately read for optical density at 405 nm (OD_405_) on a LT-4500 plate reader (Labtech). All measurements were performed in duplicate, and control wells consisting of venom-naïve sheep IgG or horse F(ab’)_2_ (diluted 1 in 25 or 1 in 5 in PBS respectively to match average protein concentration of the antivenoms at 1 in 500 dilution, then serial diluted as per antivenom), as well as secondary antibody only, were also included.

PLA_2_ assay

All test conditions were assayed in triplicate for all plates, and plates were measured on a CLARIOStar (BMG Biotech) at excitation 485-15 nm and emission 520-10 nm. For all data analyses, buffer only well values were subtracted from all other values as per manufacturer instructions. A bee venom PLA_2_ standard curve (provided in the assay kit) was ran in each assay plate in accordance with manufacturer instructions to determine the specific PLA_2_ enzymatic activity, and the specific activity of test conditions were determined by interpolation from the equation of the bee venom PLA_2_ standard curve (plotted in Prism 9, GraphPad, RRID:SCR_002798). Optimisation of the amount of venom to be used for each species was first performed to identify the amount of venom that falls within the linear range of enzymatic activity measurements. The relative fluorescence units (RFU) were plotted against the amount of venom per well, and the graphs were manually assessed to identify venom amounts in the linear range of the assay. From these results, the optimal venom amounts were determined as 1 µg for E. romani (Cameroon), *E. ocellatus* (Ghana) and *E. romani* (Nigeria). RFU was converted to specific PLA_2_ enzymatic activity (U/mL/µg) in Excel (Microsoft, RRID:SCR_016137) using the equation derived from the bee venom PLA_2_ standard curve to compare the PLA_2_ activity of the venoms. Statistical analysis of activity was by ordinary one-way ANOVA performed in Prism 10 (GraphPad, RRID:SCR_00279) and Tukey’s multiple comparison post-hoc test was performed on pairwise comparisons.

Antivenoms were serial diluted two-fold (in PBS containing the pre-defined amount of venom to 12.5 µL volume per well) in a clear, polystyrene 384-well plate (Greiner BioOne #781101). Final volumes of antivenom in respective wells were 0.10 µL to 6.25 µL. Plates were incubated at 37 °C for 30 minutes then cooled to room temperature, following which 12.5 µL PLA_2_ substrate (reconstituted as per manufacturer instructions) was added to each well. Plates were incubated in the dark at room temperature for 10 minutes and then read in a CLARIOstar plate reader (BMG Labtech). RFU measurements were converted to PLA_2_ activity using the equation of the standard curve, and then expressed as percentage of activity (where the venom only control was 100% activity) using Microsoft Excel (Microsoft, RRID:SCR_016137). For statistical analyses the data were analysed using two-way ANOVA (multiple comparisons) in Prism 9 (GraphPad, RRID:SCR_002798) to compare the antivenoms at each dilution.

SVMP assay

1 µL of 500 ng/µL venom or equal volume of PBS was added to each well in a clear, polystyrene 384-well plate (Greiner Bio-One), followed by 10 µL of antivenom (at dilutions of 1 in 4, 1 in 8, 1 in 16 and 1 in 32 equating to 2.5, 1.25, 0.625 and 0.313 µL/well) or an equal volume of PBS. Venom only, antivenom only and PBS only controls were included. The 6.2 mM SVMP substrate ES010 (BioTechne) was diluted in reaction buffer (150 mM NaCl, 50 mM Tris-HCl pH 7.5) to a 7.86 µM substrate solution. The assay plate was incubated at 37 °C for 25 minutes and then placed at room temperature for 5 minutes before the addition of 90 µL SVMP substrate solution to each well (7 µM final well concentration in the final well volume of 101 µL). The plate was immediately read at excitation 320-10 nm and emission 420-10 nm with automatic gain for 75 minutes on a CLARIOstar plate reader (BMG Labtech). All conditions were performed in replicates of four within the plate. For analysis, the RFU at 60 minutes was analysed. SVMP activity was calculated for each venom, in which ‘venom only’ wells represent 100% activity and the change in SVMP activity in the presence of the test antivenoms was calculated as a percentage of the ‘venom only’ wells. Ordinary one-way ANOVA was performed in Prism 9 (GraphPad, RRID:SCR_00279) and Tukey’s multiple comparison post-hoc test was performed on pairwise comparisons.

Plasma clotting assay

1 µL of 100 ng/µL venom or equal volume of PBS was added to each well in a clear, polystyrene 384-well plate (Greiner Bio-One), followed by 10 µL of antivenom (at dilutions of 1 in 4, 1 in 8, 1 in 16 and 1 in 32 equating to 2.5, 1.25, 0.625 and 0.313 µL/well) or equal volume of PBS. Venom only, antivenom only and PBS only controls were also included, and all conditions were performed in replicates of four within the plate. The assay plate was incubated at 37 °C for 25 minutes then room temperature for 5 minutes, before 20 µL of 20 mM calcium chloride (Sigma, #C1016) followed by 20 µL of citrated bovine plasma (Biowest, VWR #S0260) was added to each well. The optical density was immediately read at a wavelength of 595 nm (OD_595_) for 115 minutes on a CLARIOstar plate reader (BMG Labtech). For analysis, the cross-section at which the ‘normal plasma clotting’ curve intersected the curves of the test conditions was manually identified and the area under the curve at this time point for each condition was calculated (normalised to venom and PBS only controls) before converting to percentage activity as described in the SVMP assay in Section 2.7. Ordinary one-way ANOVA was performed in Prism 9 (GraphPad, RRID:SCR_002798), and Tukey’s multiple comparison post-hoc test was performed on pairwise comparisons.

Preclinical assays

Holding room conditions were 23°C with 45-65% humidity and 12/12 hour light cycles (350 lux). Mice were housed in Techniplast GM500 cages (floor area 501 cm^2^) containing 120 g Lignocell wood fibre bedding (JRS, Germany), Z-nest biodegradable paper-based material for nesting and environmental enrichment (red house, clear polycarbonate tunnel and loft). Mice had *ad libitum* access to irradiated PicoLab food (Lab Diet, USA) and reverse osmosis water in an automatic water system. Cages were changed fortnightly with fresh material in the new cage. Cages were selected at random for experimental treatments, and all experiments used mixed gender experimenters who were unblinded to the test articles.

The protocols were prepared before the study with the research questions of i) determining ED_50_ of the three antivenoms against *E. romani* (Nigeria) venom and (ii) assessing the survival rates of animals injected with *E. romani* (Cameroon) and *E. ocellatus* (Ghana) when given a fixed antivenom dose.

Animals were continuously monitored throughout the experiment for symptoms of systemic venom toxicity (starred coat, grimace, hunching, slumping, decreased movement, respiration abnormalities, strength of grip and maintenance of righting reflex, body temperature) and reaching humane endpoints (HEP) (seizure, nasal haemorrhage or loss of righting reflex) [1]. The experiment length was six hours, in accordance with previous demonstrations that for *E. ocellatus s. l.* there is no statistical difference in outcomes when comparing six hour or 24-hour duration experiments[2].

References

1. Bolton FMS. INCORPORATING THE 3RS (REFINEMENT, REPLACEMENT AND REDUCTION OF ANIMALS IN RESEARCH) INTO THE PRECLINICAL ASSESSMENT OF SNAKE VENOM TOXICITY AND ANTIVENOM EFFICACY. 2017. Available: https://livrepository.liverpool.ac.uk/3007573/

2. Durán G, Solano G, Gómez A, Cordero D, Sánchez A, Villalta M, et al. Assessing a 6-h endpoint observation time in the lethality neutralization assay used to evaluate the preclinical efficacy of snake antivenoms. Toxicon X. 2021;12: 100087. doi:10.1016/j.toxcx.2021.100087
