# Supplementary figures and images for: Preclinical evaluation of the neutralising efficacy of three antivenoms against the venoms of the recently taxonomically partitioned E. ocellatus and E. romani
Source: PLoS Negl Trop Dis. 2025 Aug 4;19(8):e0013371. doi: 10.1371/journal.pntd.0013371 (PMC12321083; doi:10.1371/journal.pntd.0013371)

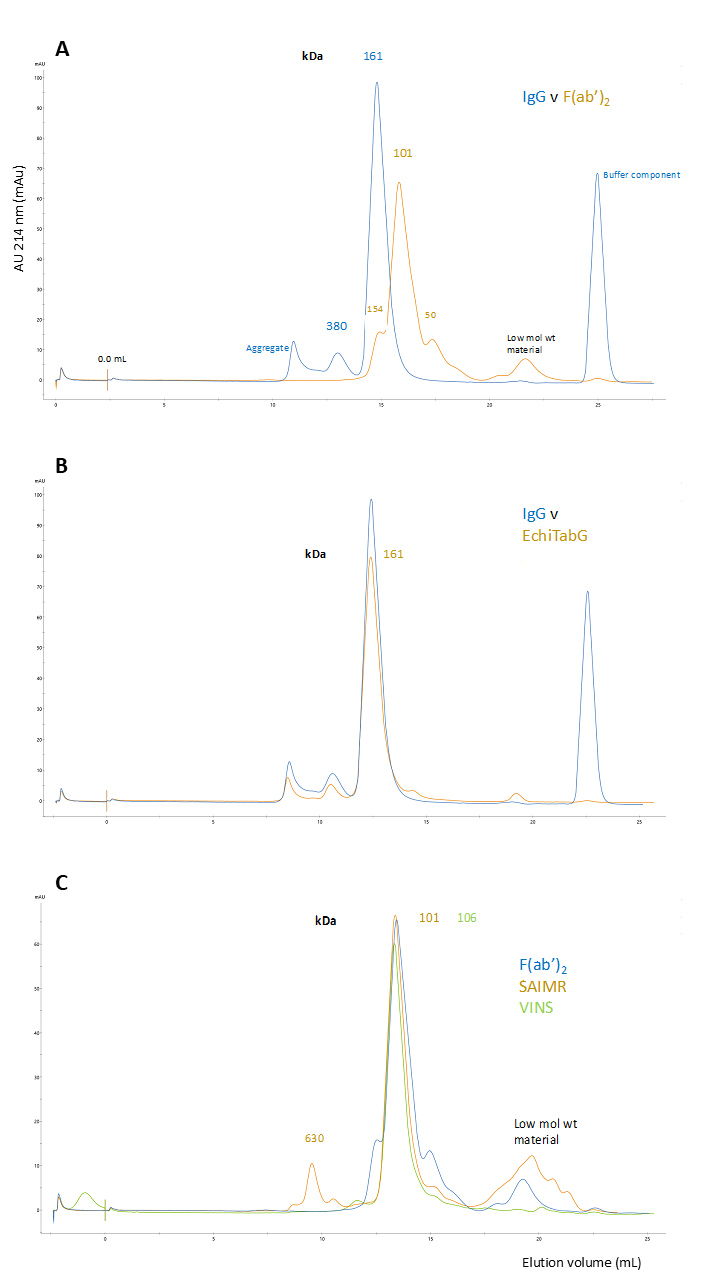

Supplement: S1 Fig — Analysis was carried out using a Superdex 200 SEC column equilibrated in PBS and elution was monitored at 214 nm. Fifty μL (50 μg) of sample was loaded. The native molecular weights (kDa) of the proteins are indicated above the respective peak.: SEC analysis of (A) control immunoglobulins whole sheep IgG (blue) and equine F(ab’)2 (gold), (B) whole sheep IgG (blue) and EchiTAbG (gold) and (C) equine F(ab’)2 (blue), SAIMR Echis (gold) and Echiven (‘VINS’, green). (TIF) [file pntd.0013371.s008.tif]

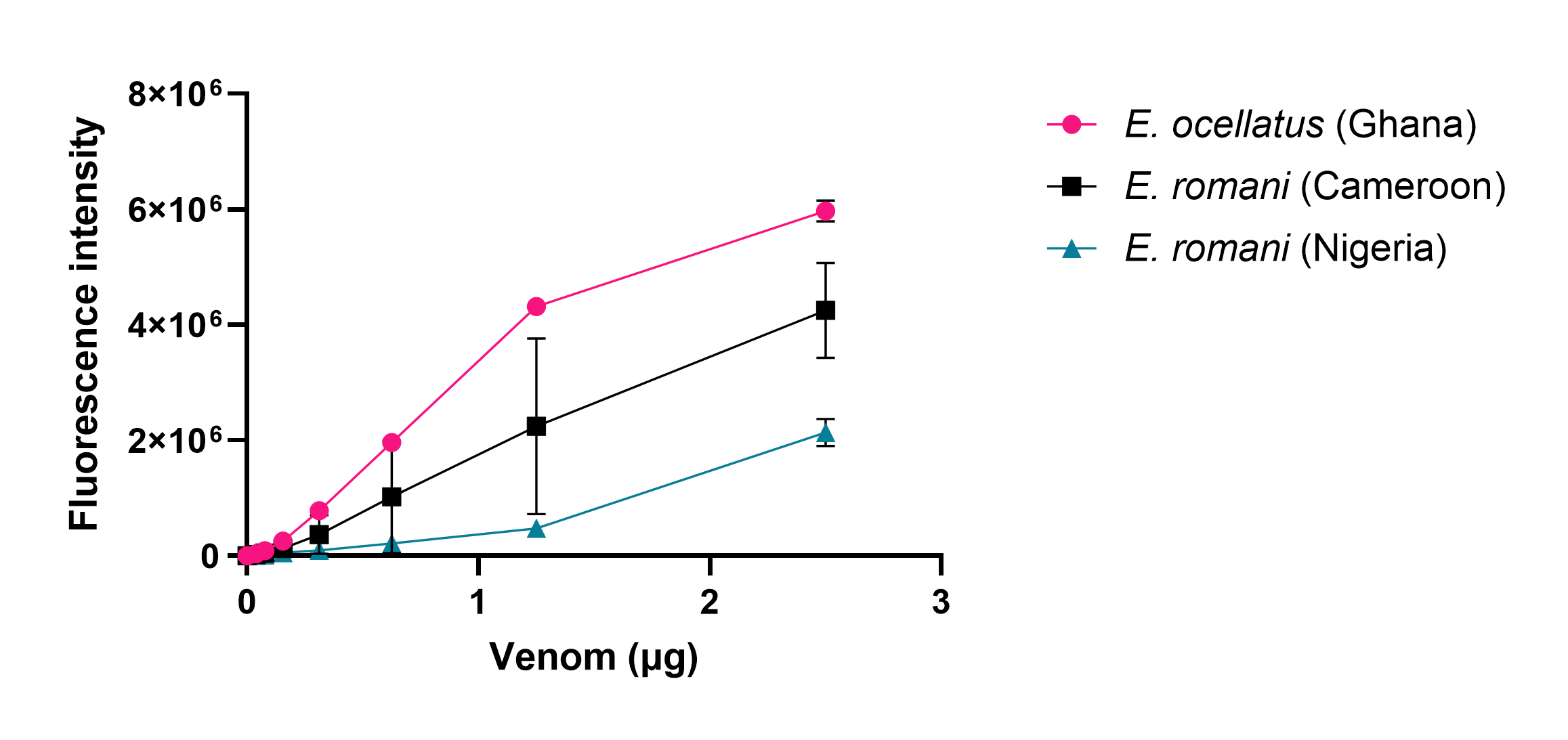

Supplement: S2 Fig — Graphs show the fluorescence intensity measured in the EnzCheck PLA2 assay with different amounts of each venom. Amounts of venom that fall in the linear range were used for subsequent assays of venom PLA2 neutralisation by antivenoms. E. ocellatus (Ghana) shown in magenta, E. romani (Cameroon) shown in black and E. romani (Nigeria) shown in teal. Samples were subtracted for background fluorescence. Data show the mean of three replicates and error bars represent standard deviation. (TIF) [file pntd.0013371.s009.tif]

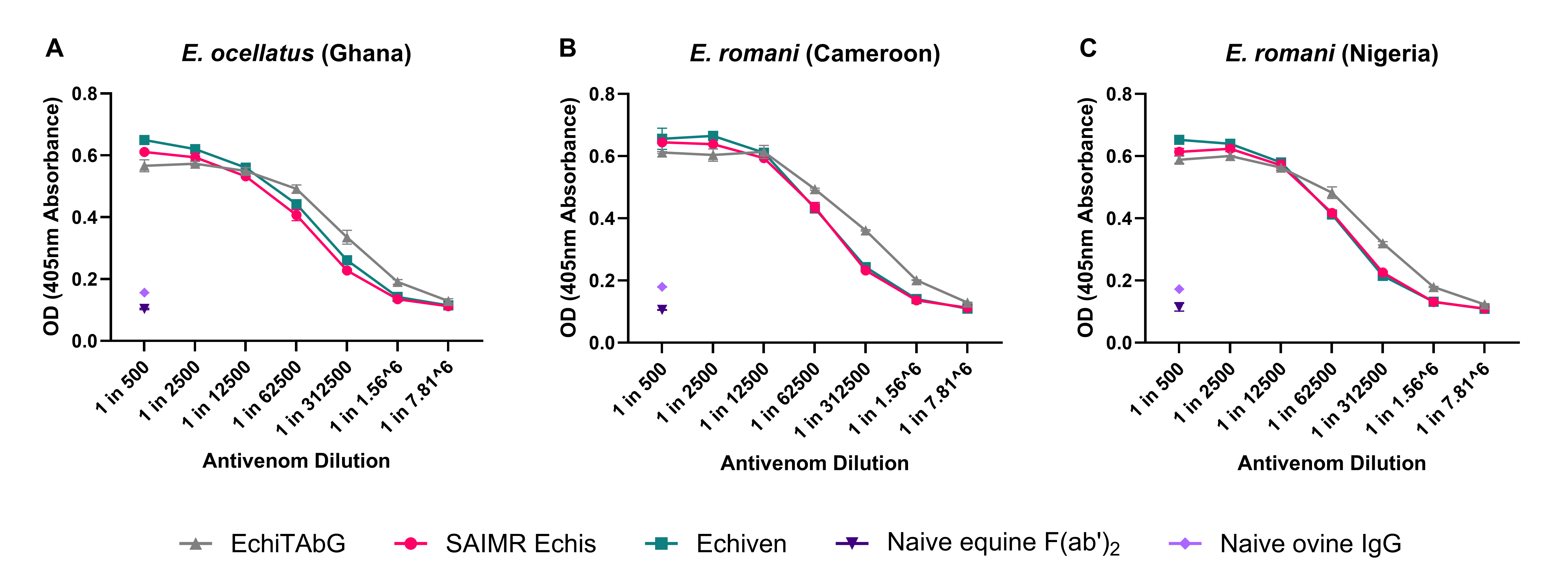

Supplement: S3 Fig — EchiTAbG shown in grey, SAIMR Echis shown in magenta, and Echiven shown in teal. Venom-naïve equine F(ab)’2 shown in dark purple and venom-naïve ovine IgG shown in light purple. Panel A: E. ocellatus (Ghana). Panel B: E. romani (Cameroon). Panel C: E. romani (Nigeria). Data points represent the mean of two replicates and error bars show the standard deviation. (TIF) [file pntd.0013371.s010.tif]
